# Cross-Kingdom RNAi of Pathogen Effectors Leads to Quantitative Adult Plant Resistance in Wheat

**DOI:** 10.3389/fpls.2020.00253

**Published:** 2020-03-10

**Authors:** Luisa Katharina Schaefer, Francis Parlange, Gabriele Buchmann, Esther Jung, Andreas Wehrli, Gerhard Herren, Marion Claudia Müller, Jonas Stehlin, Roman Schmid, Thomas Wicker, Beat Keller, Salim Bourras

**Affiliations:** ^1^Department of Plant and Microbial Biology, University of Zurich, Zurich, Switzerland; ^2^Department of Forest Mycology and Plant Pathology, Division of Plant Pathology, Swedish University of Agricultural Sciences, Uppsala, Sweden

**Keywords:** cross-kingdom RNAi, ck-RNAi, host-induced gene silencing, HIGS, ribonuclease-like effectors, effectors, *Blumeria graminis*, wheat

## Abstract

Cross-kingdom RNA interference (RNAi) is a biological process allowing plants to transfer small regulatory RNAs to invading pathogens to trigger the silencing of target virulence genes. Transient assays in cereal powdery mildews suggest that silencing of one or two effectors could lead to near loss of virulence, but evidence from stable RNAi lines is lacking. We established transient host-induced gene silencing (HIGS) in wheat, and demonstrate that targeting an essential housekeeping gene in the wheat powdery mildew pathogen (*Blumeria graminis* f. sp. *tritici*) results in significant reduction of virulence at an early stage of infection. We generated stable transgenic RNAi wheat lines encoding a HIGS construct simultaneously silencing three *B.g. tritici* effectors including *SvrPm3^*a*1/f1^*, a virulence factor involved in the suppression of the *Pm3* powdery mildew resistance gene. We show that all targeted effectors are effectively downregulated by HIGS, resulting in reduced fungal virulence on adult wheat plants. Our findings demonstrate that stable HIGS of effector genes can lead to quantitative gain of resistance without major pleiotropic effects in wheat.

## Introduction

RNA interference (RNAi) is a biological process in which small non-coding RNAs (sRNAs) are employed to selectively downregulate gene expression at the transcriptional or post-transcriptional level. Post-transcriptional gene silencing is a highly regulated mechanism relying on a cohort of proteins that direct gene silencing based on sequence complementarity between the sRNA and a target mRNA (Gheysen and Vanholme, 2007; Shabalina and Koonin, 2008). Plants encode functionally diverse populations of regulatory sRNAs which include microRNAs (miRNAs) and short interfering RNAs (siRNAs). While miRNAs correspond to 20–22-nt sequences typically derived from imperfect RNA hairpin structures, siRNAs refer to 20–24-nt sequences typically processed from long double-stranded RNA (dsRNA) precursors (Borges and Martienssen, 2015; D’Ario et al., 2017). Both siRNAs and miRNAs are processed from dsRNA precursors by the ribonuclease Dicer, and both can regulate gene expression. RNAi participates in the regulation of diverse biological processes including plant immunity (Brant and Budak, 2018; Deng et al., 2018), and several siRNAs and miRNAs have been described as important players in plant defense against viruses, bacteria, and fungi (Hua et al., 2018; Rosa et al., 2018; Muhammad et al., 2019).

Small non-coding RNAs can be expressed in a tissue-specific or stage-specific manner (Mohorianu et al., 2011; Mao et al., 2012) and correspond to highly mobile molecules that can travel from cell-to-cell (short-range) or systemically (long-range) in the plant (Dunoyer et al., 2013). There is increasing evidence demonstrating that sRNAs are also mobilized in bi-directional exchanges between plants and their parasites, thus providing a basis for cross-kingdom RNAi (ck-RNAi) as a plant defense mechanism (Knip et al., 2014; Hua et al., 2018). Prominent examples include miRNAs from the parasitic plant *Cuscuta campestris*, and siRNA *Bc-siR37* from the fungal pathogen *Botrytis cinerea*, which act as virulence factors downregulating several *Arabidopsis thaliana* genes involved in immunity (Weiberg et al., 2013; Wang et al., 2017; Shahid et al., 2018). This phenomenon can be partially explained by the fact that the three proteins that are important for RNAi, an argonaute protein, a dicer-like (DCL) protein, and a RNA-dependent RNA polymerase, are commonly found across eukaryotes (Shabalina and Koonin, 2008). While experimental evidence for the transfer of sRNAs from fungi to their host has been provided recently (Weiberg et al., 2013; Wang et al., 2017), sRNA mobility from plant to fungi has been used for host-induced gene silencing (HIGS) for almost a decade (Nowara et al., 2010; Qi et al., 2019). In plant-pathogen interactions, HIGS can be based on the uptake of exosome-like vesicles, containing transgene-derived siRNAs from the host (Cai et al., 2018).

In the obligate biotrophic cereal powdery mildew *formae speciales* (*Blumeria graminis* ff. spp.), HIGS is an important tool for functional genomics (Nowara et al., 2010; Pliego et al., 2013). In this system, successful infection is typically characterized by the formation of a highly specialized feeding structure called the haustorium, shortly after an appressorium-mediated penetration of the plant cell wall (Praz et al., 2018). The haustorium is a poorly understood membrane invagination that develops inside the host epidermal cell, and serves as a basis for the emergence of a network of strictly epiphytic hyphae which can form secondary appressoria (Kwaaitaal et al., 2017). The latter will then infect the same cell or the surrounding cells and form additional haustoria. These invasive structures are the sites of molecular exchange between *B. graminis* and its hosts. In particular, it was demonstrated that haustoria are the main interface for the delivery of candidate small secreted effector proteins (CSEPs) which promote pathogen virulence (Bourras et al., 2018).

The *B. graminis* genomes encode one of the largest repertoires of effectors in fungi, consisting of over 800 annotated proteins (Bourras et al., 2018; Frantzeskakis et al., 2018; Müller et al., 2019). Transient HIGS has been successfully used to functionally validate such candidate virulence factors in barley powdery mildew (*B. graminis* f. sp. *hordei*), leading to the identification of 21 *bona fide* effectors involved in host penetration or promoting haustorium formation (Zhang et al., 2012; Pliego et al., 2013; Ahmed et al., 2015, 2016). Of these, HIGS of the *B.g. hordei* effectors BEC1054 and BEC1011 resulted in a 60–70% reduction of haustorium formation, suggesting that some *B. graminis* CSEPs are probably essential for virulence (Pliego et al., 2013). Further molecular and biochemical characterization of these HIGS-assayed effectors revealed that they are interacting with host proteins involved in plant immunity (reviewed in Bourras et al., 2018). Relevant examples include CSEP0055 which interacts with the barley pathogenesis-related protein PR17c involved in resistance to penetration (Zhang et al., 2012), and the ribonuclease-like CSEP0064/BEC1054 which targets several barley proteins implicated in defense responses (Pennington et al., 2016, 2019). Based on these results, it has been suggested that HIGS of a few essential *B. graminis* effectors via stable host transformation could lead to a significant to permanent loss of pathogen virulence.

In the recent decade, ck-RNAi has emerged as a possible approach to control diseases in crops (Koch and Kogel, 2014; Hua et al., 2018). Several studies have described the use of stable HIGS in plants to confer resistance to fungal pathogens (Nowara et al., 2010; Koch et al., 2013; Chen et al., 2016; Panwar et al., 2017; Guo et al., 2019). Based on experimental evidence from transient assays in barley, a preliminary study using stable transgenics showed that HIGS of the *B.g. hordei 1,3-β-glucanosyl-transferase (GTF1)* gene, resulted in a decrease of fungal virulence on barley T1 seedlings (Nowara et al., 2010). So far, fungal house-keeping genes and pathogenesis-related genes were the primary gene targets for stable HIGS. In *B. graminis*, the discovery of *bona fide* effectors such as BEC1054 and BEC1011 raised the question whether stable silencing of such CSEPs would be equivalent to silencing an essential gene. In the BEC1054 and BEC1011 HIGS assays, single barley epidermal cells were biolistically transformed with a HIGS construct encoding a long dsRNA sequence, perfectly complementary to a segment of the effector mRNA sequence (Pliego et al., 2013). *B.g. hordei* virulence was scored microscopically at the haustorial stage, based on the number of formed haustoria, normalized to the number of penetration attempts (Pliego et al., 2013). Because of the technical limitation of this strategy, it was not possible to quantify target gene expression or to assess macroscopic phenotypes. In this context, HIGS of effectors using stable transgenic lines would be an opportunity to understand the impact of ck-RNAi on pathogen virulence, based on accurate, whole tissue assessment of target gene silencing in relation to plant resistance.

Here, we demonstrate the applicability of HIGS in *B.g. tritici* based on the silencing of the *β2-tubulin (β2-tub)* housekeeping gene. We also present the generation, selection, and characterization of stable wheat lines expressing a HIGS construct targeting the suppressor of avirulence gene *SvrPm3^*a*1/f1^*. This wheat powdery mildew effector is involved in the suppression of several allelic *Pm3* resistance gene variants in wheat (Bourras et al., 2015, 2019; Parlange et al., 2015). We also show that *SvrPm3^*a*1/f1^* is expressed at significantly lower levels in *B.g. tritici* colonies growing on the HIGS lines as compared to the non-transgenic control. Consistent with *in silico* prediction of possible targets in the pathogen, two additional members of the *SvrPm3^*a*1/f1^* effector gene family sharing sequence homology are also downregulated by HIGS. Finally, based on extensive phenotypic characterization of infected wheat material from laboratory and semi-field experiments, we show that HIGS of *SvrPm3^*a*1/f1^* results in a quantitative gain of resistance against *B.g. tritici*.

## Results

### Establishing HIGS of *B.g. tritici* Genes in Wheat

**FIGURE 1:**
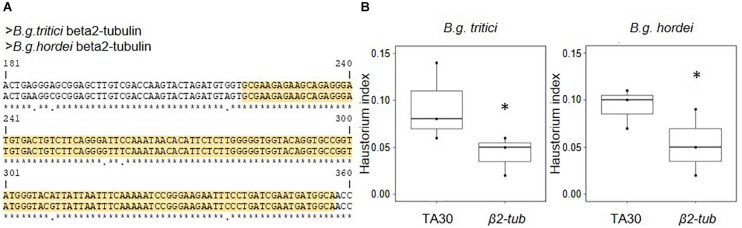
Transient HIGS of fungal β*2-tubulin* (*β2-tub*) reduces virulence in *B.g. hordei* and *B.g. tritici*. The *β2-tub-*RNAi construct was transiently expressed in barley and wheat epidermal cells, which were infected with *B.g. hordei* and *B.g. tritici*, respectively. **(A)** Alignment of the *B.g. tritici* and *B.g. hordei β2-tub* gene sequences. Only the fragment including the donor sequence for the *β2-tub*-RNAi construct (yellow) is depicted. The *B.g. hordei* sequence was used to construct the *β2-tub*-RNAi plasmid. **(B)** Effect of the *β2-tub*-RNAi construct on virulence in both *formae speciales*. The effect on fungal virulence was measured 2 days post infection, by scoring the ratio of successful over total infection attempts (haustorium index). Results were compared to the empty vector control pIPKTA30 (TA30) using a proportion test. ^∗^*p* < 0.05.

Host-induced gene silencing of *B. graminis* genes was originally established in barley (Nowara et al., 2010). Here, we aimed at establishing HIGS in wheat for targeting *B.g. tritici* genes and at comparing HIGS efficacy in wheat and barley powdery mildew. The *B. graminis β2-tub* gene, an essential housekeeping gene and a canonical fungicide target (Zhou et al., 2016; Vela-Corcía et al., 2018), was selected as target gene for this assay. There is no *β1-tub* gene in *B. graminis*, indicating that transcriptional knockdown of the single-copy *β2-tub* gene is highly relevant for assessing the impact of HIGS on fungal virulence. Due to high sequence similarity between *β2-tub* genes across *B. graminis* ff. spp., the sequence from *B.g. hordei* was used as a template to design a HIGS construct capable of silencing this gene in *B.g. tritici* and *B.g. hordei* (Figure 1A). The silencing construct is based on a 140-nt segment of exon 6 from the *B.g. hordei β2-tub* gene (Supplementary File 1). The *β2-tub-*RNAi sequence used in this study was analyzed for possible off-targets in both *formae speciales* and their host species wheat and barley using the si-Fi software, which predicted no off-targets in either of the analyzed *B. graminis* and host genomes (Lück et al., 2019).

Wheat and barley leaves were biolistically co-transfected with a GUS reporter plasmid and the *β2-tub*-RNAi plasmid and infected with *B.g. tritici* and *B.g. hordei*, respectively. Fungal virulence was assessed microscopically 2 days post infection (dpi) based on successful haustoria formation (haustorium index, see section “Materials and Methods”). The empty pIPKTA30 vector was used as negative control. Results from three independent biological replicates showed statistically significant reduction of fungal virulence, corresponding to a decrease of the haustorium index by −42% in *B.g. tritici* and −43% in *B.g. hordei* as compared to the negative control (Figure 1B). We observed no effect on hyphae formation in the successful colonies. We conclude that transient HIGS of an essential housekeeping gene in *B.g. tritici* and *B.g. hordei* leads to very similar, quantitative loss of virulence in both *formae speciales*. Thus, HIGS studies are feasible in wheat using the same vector design and phenotyping strategy. We suggest that targeting of a housekeeping gene, such as *β2-tub*, can be used as a technical reference for assessing the relative efficiency of HIGS in future assays.

### Generation of Stable *SvrPm3^*a*1/f1^*-RNAi Wheat Lines

**FIGURE 2:**
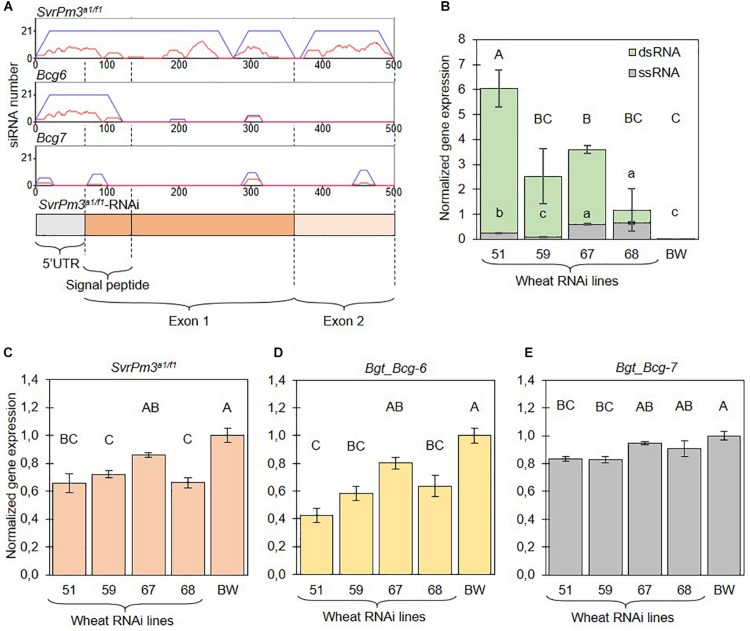
The *SvrPm3^*a*1/f1^*-RNAi transgene is constitutively expressed in transgenic wheat lines and silences three *B.g. tritici* effectors. **(A)** si-Fi software predictions of *SvrPm3^*a*1/f1^*-RNAi transgene-derived siRNAs that are efficient (red lines) or inefficient (blue lines) in directing silencing of the three putative target effectors *SvrPm3^*a*1/f1^*, *Bgt_Bcg-6*, and *Bgt_Bcg-7.* On the y-axis is the number of siRNAs that bind to a given locus on the x-axis. **(B)** Quantification of transgene-derived ssRNA and dsRNA transcripts from non-infected samples. **(C–E)** Quantification of target gene transcripts from infected samples. Samples were collected 3 days post infection. Error bars represent the standard error. Statistical difference was tested using ANOVA **(B:**dsRNA,**D,E)** or Welch ANOVA **(B:**ssRNA,**C)**, according to the data distribution and variance. Different letters indicate statistical significance.

We designed a new HIGS construct targeting the *B.g. tritici* effector *SvrPm3^*a*1/f1^*. We selected a 500-nt fragment of the *SvrPm3^*a*1/f1^* mRNA, which includes a segment of the 5′UTR and both exons excluding the last two codons (Figure 2A). Off-target analysis using the si-Fi software (Lück et al., 2019) predicted four putative siRNAs that map to a single 30-nt off-target locus in a putative pseudogene on the wheat chromosome 6A. However, these siRNAs are predicted to be inefficient in directing silencing (Supplementary Note 1 and Supplementary File 2). Off-target analysis using the *B.g. tritici* genome identified two additional target genes, *Bgt_Bcg-6* and *Bgt_Bcg-7* (Figure 2A and Supplementary Table 1). Consistent with the off-target predictions, both effector genes share nucleotide homology with *SvrPm3^*a*1/f1^*, with *Bgt_Bcg-6* being identical in the 5′ first 120-nt of the HIGS construct (Supplementary Figure 1). *Bgt_Bcg-6* and *Bgt_Bcg-7* both belong to the *SvrPm3^*a*1/f1^* effector gene family and are the closest homologs of the target effector (Parlange et al., 2015; Müller et al., 2019). Based on these results, we conclude that *SvrPm3^*a*1/f1^*, *Bgt_Bcg-6*, and *Bgt_Bcg-7* are potent targets of the HIGS construct designed for the generation of stable transgenics.

We cloned the selected *SvrPm3^*a*1/f1^*-RNAi sequence into the pIPKb007 vector, in which sense and antisense sequences are separated by a wheat *RGA2* intron allowing hairpin formation (Himmelbach et al., 2007). Transgene expression is controlled by the maize ubiquitin promoter and the CaMV 35S terminator (Supplementary Figure 2). The spring wheat cultivar Bobwhite SH 98 26 (BW) was co-transformed with the linearized HIGS construct and the *phosphomannose isomerase* gene (*Pmi*) as a selection marker by particle bombardment. We obtained 138 independent T0 transformants (events), which were propagated to the T1 generation. Macroscopic scoring of leaf coverage by *B.g. tritici* colonies in T1 seedling leaves, did not allow us to discriminate between the segregating transgenic and non-transgenic T1 progeny. We hypothesized that our assay was not sensitive enough for scoring quantitative effects. We therefore aimed at selecting four events with the following criteria (i) 1–2 copies of the transgene can be detected by southern blot (Supplementary Figure 3), (ii) both the sense and antisense copy of the HIGS construct can be amplified by PCR indicating that the complete HIGS transgene is integrated into the genome, (iii) events are homozygous, (iv) transgene expression can be detected by RT-PCR, and (v) no major pleiotropic effects on plant development resulting from tissue culture can be observed. Based on this combination of criteria, we could confidently select four events, #51, #59, #67, and #68. All are single copy events, except event #67 which contains two genetically linked transgene copies.

We conclude that the generation of stable transgenic HIGS wheat plants can be restricted by the following challenges: First, T1 plants are difficult to phenotype for quantitative, small effects in which replication and scalability are key for inferring statistically significant differences. Second, the hairpin secondary structure inherent to the design of the HIGS construct complicates the molecular detection of transgene copies, both at the DNA and cDNA levels, using PCR-based methods. Considering these technical limitations and our selection process in which only events that pass all selection criteria are further characterized, we suggest that we are probably underestimating the proportion of successful transformation events.

### The *SvrPm3^*a*1/f1^*-RNAi Transgene Is Constitutively Expressed

In recent work by Wang et al. (2018), it was demonstrated that standard RT-qPCR assays are not suitable for the quantification of dsRNA transcripts produced by RNAi transgenes. Standard protocols for mRNA quantification by RT-qPCR primarily quantify aberrant single-stranded RNA (ssRNA) transcripts, which are inefficient in directing gene silencing (Wang et al., 2018). To further characterize the four selected events, we adapted the protocol described by Wang et al. (2018) and quantified dsRNA and ssRNA transcripts in non-infected seedlings. Consistent with previous findings by Wang et al. (2018), our assays show that expression of dsRNA and ssRNA transcripts are not correlated, thus further corroborating the importance of validating dsRNA expression from RNAi transgenes. But most importantly, we found that dsRNA transcripts are generally more abundant than ssRNA transcripts thus demonstrating that the HIGS transgene is giving rise to a proper template for siRNA biogenesis (Figure 2B). Together this data demonstrate that all four events contain a full, transcriptionally active copy of the *SvrPm3^*a*1/f1^*-RNAi transgene, which is constitutively expressed in absence of *B.g. tritici* infection.

We conclude that our selection procedure, based on careful molecular validation of transgene integrity at the DNA and RNA levels, led to the identification of relevant events, constitutively expressing the HIGS transgene. In this context, we propose that this material is ideal for assessing the effect of stable HIGS on mildew virulence at different stages of wheat development.

### The *SvrPm3^*a*1/f1^*-RNAi Transgene Reduces Target Effector Gene Expression

The *B.g. tritici* effectors *SvrPm3^*a*1/f1^*, *Bgt_Bcg-6*, and *Bgt_Bcg-7* are equally valid potential targets of the *SvrPm3^*a*1/f1^*-RNAi transgene (Figure 2A). To test whether the three targets are effectively silenced, we quantified mRNA expression from *B.g. tritici* infected seedlings at three dpi, a time point corresponding to the haustorial stage. Previous studies have shown that gene expression levels of *SvrPm3^*a*1/f1^* are the highest during haustorium formation (Bourras et al., 2015, 2019; Praz et al., 2018). Similarly, several members of the *SvrPm3^*a*1/f1^* effector gene family, including *Bgt_Bcg-6* and *Bgt_Bcg-7*, are induced at that same stage (Supplementary Figure 4) (Praz et al., 2018; Bourras et al., 2019), suggesting that this time point is particularly appropriate for the quantification of mRNA levels of all three effectors, and to assess HIGS efficiency. Transgenic events and wildtype BW, were infected with the *B.g. tritici* isolate Bgt_JIW2, which shows intermediate levels of expression of *SvrPm3^*a*1/f1^* (Bourras et al., 2015, 2019). Primer design for specific amplification of the respective fungal *SvrPm3^*a*1/f1^*, *Bgt_Bcg-6*, and *Bgt_Bcg-7* mRNAs was challenging, as the sequences of the three effectors are rather similar, and since the transgene corresponded to almost the complete *SvrPm3^*a*1/f1^* coding sequence. Therefore, RT-qPCR primers could only be designed on the 3′UTR to discriminate between the transgene, *SvrPm3^*a*1/f1^* and *Bgt_Bcg-6* in particular. Specificity was verified by Sanger sequencing of amplicons from infected leaf material and non-infected controls.

The mRNA levels of the target genes *SvrPm3^*a*1/f1^* and *Bgt_Bcg-6* were significantly reduced as compared to the wildtype BW control in all events except #67 (Figures 2C,D). Interestingly, silencing of *Bgt_Bcg-6* was consistently similar and sometimes even more effective than silencing of *SvrPm3^*a*1/f1^* (Figures 2C,D). For *Bgt_Bcg-7*, we observed a less pronounced but yet significant reduction of mRNA levels in events #51 and #59, but not in event #68 (Figure 2E). Here, differences in target gene silencing are in agreement with the differences in sequence similarity between the three effectors. In particular, we propose that the 120-nt segment that is identical between *SvrPm3^*a*1/f1^* and *Bgt_Bcg-6* is possibly responsible for the silencing of both genes, while lower sequence identity between these two effectors and *Bgt_Bcg-7* in that same segment is likely responsible for reduced HIGS efficiency on the latter. All effector targets considered, target gene downregulation ranged from −16% (*Bgt_Bcg-7* on #51) to −58% (*Bgt_Bcg-6* on #51), thus molecularly demonstrating that the HIGS transgene is functional in the events #51, #59 and #68, and does mediate ck-RNAi.

We conclude that stable wheat lines constitutively expressing a HIGS transgene can effectively mediate target gene silencing in *B.g. tritici*. We also conclude that the 5′ 120nt of the HIGS construct are probably sufficient for achieving high levels of target silencing, which could also be combined with other RNAi sequences for simultaneous silencing of entire gene families to potentially strengthen the impact of HIGS.

### Effector Silencing Results in an Event-Specific Quantitative Loss of Virulence

**FIGURE 3:**
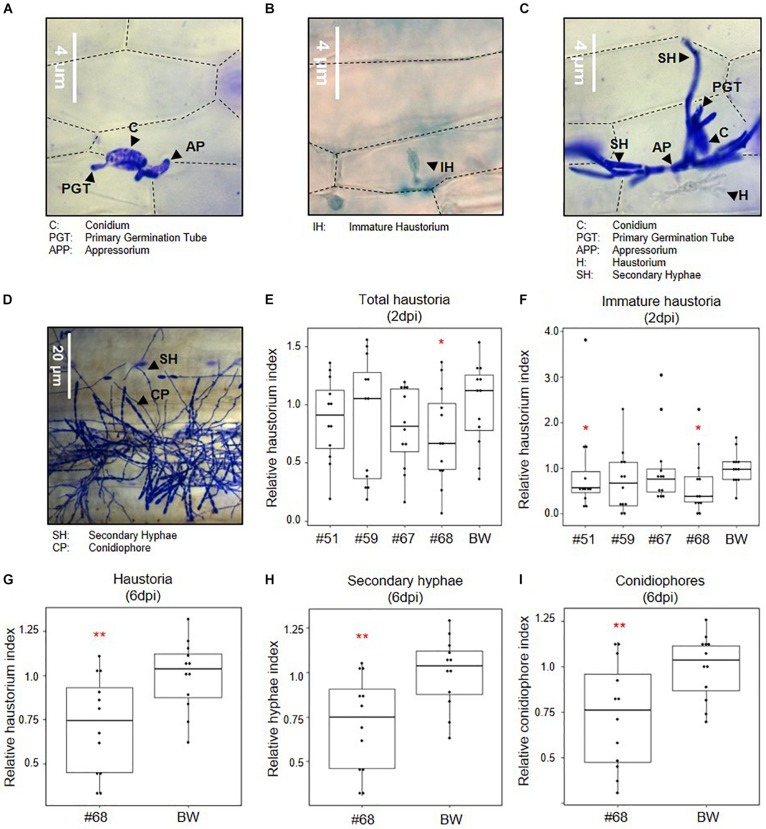
Effect of effector gene silencing on infection success. Seedlings of *SvrPm3^*a*1/f1^*-RNAi wheat lines were infected with *B.g. tritici*, and virulence was scored at two- and six-days post infection (dpi). All data is normalized to the wildtype control Bobwhite (BW). **(A–D)** Representative pictures of *B.g. tritici* developmental stages. **(A)** A spore has formed an appressorium, but fails at infection. **(B)** An immature haustorium. **(D)** A successful colonization at ca. two dpi with a mature haustorium and secondary hyphae. **(D)** A successful *B.g. tritici* colony at six dpi with mature conidiophores. **(E)** Total haustorium index at two dpi. **(F)** Immature haustorium index at two dpi. **(G)** Haustorium index at six dpi. **(H)** Hyphae index at six dpi. **(I)** Conidiophore index at six dpi. Pairwise comparison of transgenic events to the wildtype BW was carried out using a one-sided *t*-test **(E,G–I)** or a Wilcoxon rank sum test **(F)**, according to the data distribution and variance. *p < 0.05; **p < 0.01.

Based on the functional validation of target gene silencing, the impact of simultaneous silencing of three effectors, *SvrPm3^*a*1/f1^*, *Bgt_Bcg-6*, and *Bgt_Bcg-7*, on *B.g. tritici* virulence was assessed at different developmental stages (Figures 3A–D). First, we infected leaf segments from seedlings of the transgenic events and the wildtype control with the *B.g. tritici* isolate Bgt_JIW2 using a low-density inoculum, and quantitatively scored virulence based on the infected leaf area at six dpi. In this assay, we observed no reduction of virulence of *B.g. tritici* on seedlings (Supplementary Figure 5). We hypothesized that the effect of HIGS is best observed at the haustorial stage when the target effectors, in particular *SvrPm3^*a*1/f1^* are transcriptionally active (Bourras et al., 2015, 2019; Praz et al., 2018). So, we prepared infected leaf segments for microscopy to score virulence at two dpi. Interestingly, we found a significant reduction in the haustorium index of −46% in event #68 compared to the wildtype (Figure 3E). The effect was stronger on the subgroup of immature haustoria, which are not yet showing the fully branched structure typical of mature haustoria (Figures 3A–C). For these, we found a significant reduction in the haustorium index of −41% in event #51 and −60% in event #68 compared to the wildtype (Figure 3F). The reduction of haustorium indices in the events #59 and #67 were not significant (Figures 3E,F). These results suggest that silencing of the three target effectors impairs *B.g. tritici* virulence at the haustorial stage in an event-specific, quantitative manner. Next, we microscopically assessed *B.g. tritici* development at four and six dpi on line #68, as it shows the strongest effect at the haustorial stage. Here, we scored *B.g. tritici* virulence by assessing the presence of haustoria, hyphae, and conidiophores. We found no significant differences at four dpi (Supplementary Figures 6C–H). However, we observed a significant reduction of the haustorium index of −30% in event #68 at six dpi compared to wildtype (Figure 3G), although no macroscopic differences in infected leaf area were observed in the initial seedling infection assays at this time point. Congruently, no additive effect was observed on hyphae and conidiophore formation (Figures 3D,H,I and Supplementary Figures 6A,B,I). Altogether, these results indicate that there is a consistent quantitative effect from HIGS of the three target effectors specifically impairing primary haustoria formation.

We conclude that the observed effects of HIGS on *B.g. tritici* development on seedlings, is in agreement with previous evidence suggesting that the effectors *SvrPm3^*a*1/f1^*, *Bgt_Bcg-6*, and *Bgt_Bcg-7* are important virulence factors particularly active at the haustorial stage. We also conclude that the observed effect is quantitative and event-specific, which could be due to genomic location of the transgene and subsequent tissue-specific variations of transgene expression.

### *SvrPm3^*a*1/f1^*-RNAi Transgenic Wheat Shows Quantitative Adult Plant Resistance to *B.g. tritici* in Semi-Field Conditions

In a final phenotypic analysis, we characterized all events for susceptibility to *B.g. tritici* at the adult stage in a semi-field setup. Here, the plants were exposed to local climate, and the local *B.g. tritici* population, We monitored *B.g. tritici* disease development over time according to Brunner et al. (2011) and calculated the area under the disease progression curve (AUDPC). We additionally collected representative samples for each test plot of flag leaves (F0), F-1 and F-2 leaves for precise quantification of infected leaf area. F0 leaves were sampled at the beginning of flag leaf infection at 70 days post sowing (dps), concurrent with spike emergence, and a second time at the end of the *B.g. tritici* disease progression at 92 dps, concurrent with seed ripening. F-1 and F-2 leaves were sampled at 78 dps.

**FIGURE 4:**
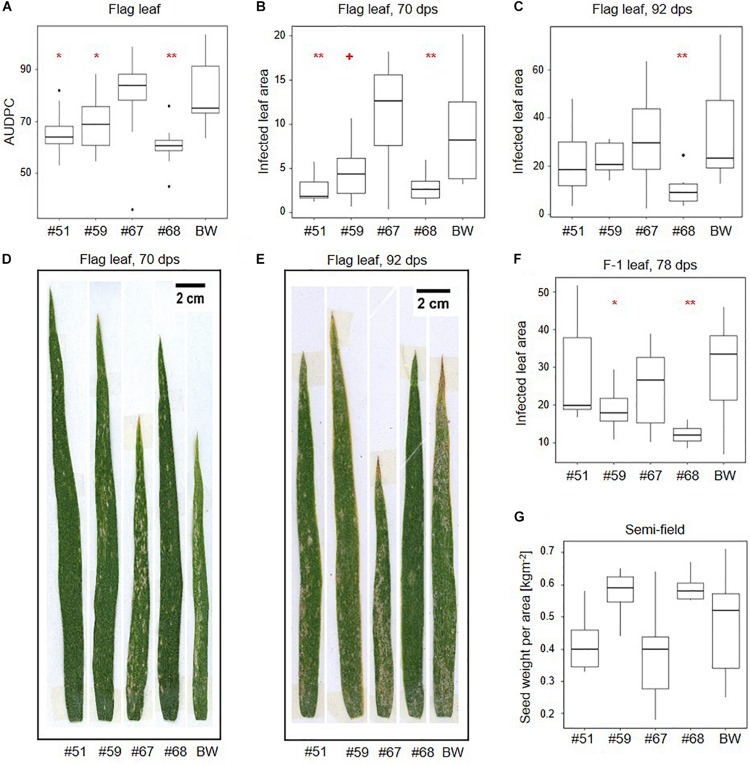
*SvrPm3^*a*1/f1^*-RNAi wheat lines show quantitative adult plant resistance in semi-field conditions. Transgenic events and wild-type Bobwhite (BW) were exposed to natural *B.g. tritici* infection in a semi-field trial. **(A)** Disease was monitored over time on the flag leaves and the area under the disease progression curve (AUDPC) was calculated. **(B–F)** Image-based quantification of the infected leaf area in flag and F-1 leaves. **(B,D)** At the beginning of flag leaf infection, 70 days post sowing (dps). **(C,E)** At the end of the *B.g. tritici* disease progression, 92 dps. **(F)** F-1 leaves at 78 dps. **(G)** Quantification of seed weight per area. Pairwise comparison of transgenic events with wildtype BW were carried out using one-sided *t*-test **(A)**, Welch *t*-test **(C,F,G)** or Wilcoxon rank sum test **(B)** depending on the distribution and variance of the data. + < 0.06; *p < 0.05; **p < 0.01.

Area under the disease progression curves based on all plant development stages did not indicate significant differences between the transgenic events and the wildtype control (Supplementary Figure 7A). However, AUDPCs based on flag leaves indicated significantly lower *B.g. tritici* infection than wildtype in the three events that also show target gene silencing (#51, #59, and #68) (Figure 4A). AUDPC is designed to score disease development on whole plants, but in our assay, it was only informative when considering the flag leaves. Therefore, we used image-based precise quantification of infected leaf area in the representative leaf samples to further support our results. In line with the AUDPC results, we found a significant reduction of infected leaf area in the flag leaf at 70 dps compared to wildtype in the same three events (−71% for #51, −50% for #59, and −70% for #68) (Figures 4B,D). Consistent with the results from seedling assays, this effect decreased over time to no significant difference at 92 dps with the exception of event #68, in which a significant reduction in infected leaf area (−69%) could still be observed (Figures 4C,E). The effect also decreased with the age of the leaves. While the F-1 leaves show a significant reduction in infected leaf area at 78 dps for events #59 (−36%) and #68 (−60%) (Figure 4F and Supplementary Figure 7B), there was no significant effect in the F-2 leaves (Supplementary Figures 7C,D).

We also compared leaf area from adult leaves and took measurements of physiological traits including spike emergence and flowering time during the semi-field experiment. Additionally, we scored plant height, seed set, and seed weight per area both in the semi-field experiment and under controlled greenhouse conditions. While we acknowledge the small scale of these experiments, our results indicate that while there are no significant differences in seed weight per area in the semi-field, there is a trend toward higher seed weight per area in lines #59 and #68 which correlates with lower infected leaf area in F-1 leaves (Figure 4G). Under controlled greenhouse conditions, the seed weight per area was significantly higher in all three lines that showed target gene silencing and reduced infected leaf area in the semi-field (#51, #59, and #68) (Supplementary Figure 8A). Higher seed weight per area partially correlates with significantly higher spike number per plant in event #51 and #68 (Supplementary Figure 8B). We also observed that the pooled F0, F-1, and F-2 leaves of the three events showing increased adult plant resistance have significantly larger leaf area than wildtype, which could explain the increase in seed weight per area (Supplementary Figure 8C). We observed no meaningful differences in flowering time, plant height, or seed set neither in the semi-field nor in the greenhouse (Supplementary Figures 8D–H,J). But we observed a trend toward a 2- to 3-day delay in median spike emergence in all four transgenic lines, which was significant for events #51 and #67 (Supplementary Figure 8I). Overall, these results indicate that the virulence reducing effect of the *SvrPm3^*a*1/f1^*-RNAi transgene on the *B.g. tritici* pathogen is not accompanied by a major effect on host performance.

We conclude that stable HIGS of the three *B.g. tritici* effectors resulted in a quantitative gain of powdery mildew resistance in wheat. Resistant events can impair haustorium formation on seedlings, and restrict *B.g. tritici* growth on adult leaves in a quantitative manner.

## Discussion

Transient HIGS has been an important tool for functional genomics in *B. graminis*. In this study, we showed that HIGS can be used in stable transgenic wheat plants to effectively target three *B.g. tritici* effectors. Most importantly, we show that the use of this strategy to impair pathogen virulence results in a quantitative gain of resistance to wheat powdery mildew in adult plants.

### Evidence for Stable Cross-Kingdom RNAi in Wheat Powdery Mildew

The stable transgenic events generated in this study constitutively express the *SvrPm3^*a*1/f1^*-RNAi transgene. Therefore, we could confidently assess and correlate the observed phenotypes with the actual downregulation of the target genes, which is a major improvement to single cell transient HIGS assays. In this study, three effector genes were targeted (*SvrPm3^*a*1/f1^*, *Bgt_Bcg-6*, and *Bgt_Bcg-7*) and RT-qPCR assays showed that all three are downregulated when the pathogen is growing on transgenic HIGS events as compared to the non-transgenic control. The level of target gene silencing ranged from −16 to −58%, which is similar to the levels of HIGS reported in other fungal pathogens such as *B. cinerea* on Arabidopsis and *Puccinia triticina* on wheat (Wang et al., 2016; Panwar et al., 2017). In *Fusarium graminearum*, an even higher level of target gene silencing was reported (−77 to −92%) on Arabidopsis, suggesting that HIGS efficiency is intrinsically regulated by the two organisms involved in ck-RNAi (Koch et al., 2013).

The biology behind sRNA transport from the host to the fungal cell through the haustorial interface is not well understood. In principle, the transgene-derived hairpin transcripts are processed by host DCL enzymes into siRNAs. These siRNAs are then secreted in exosome-like vesicles that can be taken up by the fungus via the haustorial interface (Cai et al., 2018). Once the siRNAs are taken up, they guide the RNA-induced silencing complex (RISC) to cleave homologous, fungal mRNA transcripts. In plants and worms, mRNA-cleavage by a siRNA-primed RISC leads to the accumulation of secondary siRNAs through a specific RNA-dependent RNA polymerase (RdRP), which uses the diced mRNA molecules as a template to synthesize secondary dsRNA precursors or directly siRNAs. The accumulation of secondary siRNAs is known as transitivity, and is thought to result in a more persistent and stable target gene silencing (Calo et al., 2012).

In fungi, transitivity has been described in the zygomycete *Mucor circinelloides* and relies on its RdRP homolog *McRdRP-2* (Calo et al., 2012). In ascomcyetes such as *Neurospora crassa* and *Aspergillus nidulans*, whole genome sequencing data indicates that the orthologs of the *McRdRP-2* gene have been lost (Torres-Martínez and Ruiz-Vázquez, 2017). Instead, ascomycetes have orthologs of *McRdRP-1*, e.g., *NcQde-1*, which are necessary for sense but not for inverted repeat transgene-triggered RNAi. It is unclear whether the ascomycete orthologs of *McRdRP-1* are capable of transitivity. In the case of the *Blumeria* clade, Kusch et al. (2018) found that this group of fungi has lost all RdRP paralogs except one single copy. It is unknown which specific RNA processing properties were retained by this single representative of the RdRP family in *Blumeria*, and how this protein is contributing to ck-RNAi. Based on our results, we hypothesize that ck-RNAi in *Blumeria* is possibly restricted by the absence of a transitivity-capable RdRP. This hypothesis provides an explanation for the relatively moderate silencing of target gene expression achieved by HIGS in this study. We propose that further characterization of the fungal RNAi machinery can provide meaningful information about the limitations and possible improvements of HIGS in *Blumeria*.

### The Molecular Basis for Effective Cross-Kingdom RNAi in Wheat Powdery Mildew

Efficient transgene-derived dsRNA accumulation *in planta* is thought to be decisive for efficient RNAi-directed control of insect pests (Wang et al., 2018). In fact, both dsRNA and ssRNA transcripts can be derived from a hairpin transgene, however, since only dsRNA is an efficient template for RNAi, it is anticipated that higher accumulation of dsRNA molecules should correlate with a higher accumulation of siRNAs, and thus higher target gene silencing efficiency. Here, we had a unique opportunity to test this hypothesis in a stable system in which we had clear evidence for HIGS. Interestingly, our results showed that the dsRNA proxy performed poorly in predicting the efficacy of ck-RNAi. While we could show that all events express a detectable dsRNA molecule, we found little correlation between ssRNA/dsRNA expression levels and target gene silencing. This could be explained by differences in transgene expression at the tissue level, which cannot be detected in our RT-qPCR assays based on RNA extraction from entire leaf segments. Here, higher levels of target gene silencing can possibly be associated with higher dsRNA expression in epidermal cells which are the only cells infected by mildew, and the primary target tissue of transient HIGS. It is also possible that dsRNA accumulation is not the only factor relevant for efficient ck-RNAi in *Blumeria*. In fungi, evidence suggests that dsRNA-derived siRNAs are the bioactive molecule driving HIGS (Cai et al., 2018), thus additional factors influencing dsRNA processing by the host DCL enzymes, siRNA packaging into exosome-like vesicles, and vesicle uptake by the fungus can have an impact on silencing efficiency, either acting as bottlenecks or enhancers. In humans, the discovery of sequence motifs that are over-represented in exosome-associated miRNAs led to the identification of specific proteins that control miRNA sorting into exosomes (Villarroya-Beltri et al., 2013). We therefore suggest that the identification of the genetic factors associated with RNA mobility from its hosts to *Blumeria* can provide an important leverage for improving HIGS efficiency. We speculate that favorable HIGS traits can be identified from the natural diversity of wheat cultivars, introduced through breeding or genome editing, and inform better HIGS transgene design.

### Evidence for a Conserved Role of Ribonuclease-Like Effectors in Wheat Powdery Mildew Virulence

Small secreted proteins with a predicted ribonuclease fold constitute the largest class of candidate effectors in the *Blumeria* genomes (Pedersen et al., 2012; Bourras et al., 2018). This RNase-like class of effectors also includes the *SvrPm3^*a*1/f1^* family which encodes all three effectors targeted by HIGS in this study. Evidence from previous transient HIGS experiments indicated that this class of effectors is highly important for *Blumeria* virulence at the early stages of infection (Pliego et al., 2013). In this study, we extensively scored several attributes of *B.g. tritici* virulence on stable *SvrPm3^*a*1/f1^*-RNAi wheat lines at the macroscopic and microscopic level. We observed that HIGS of the three RNase-like effectors, *SvrPm3^*a*1/f1^*, *Bgt_Bcg-6*, and *Bgt_Bcg-7*, leads to a consistent loss of virulence at the haustorial stage, in the form of a reduced ability of the fungus to penetrate and form such feeding structures, and to a significant reduction in infected leaf area on adult leaves. Our results thereby further substantiate the importance of this class of proteins for *Blumeria* virulence. Previous transient HIGS experiments in *B.g. hordei* also described an effect primarily on haustorial development upon targeting of effectors (Nowara et al., 2010; Zhang et al., 2012; Pliego et al., 2013; Ahmed et al., 2015; Aguilar et al., 2016).

Several families of *Blumeria* effectors, including the effector targets in this study, are specifically induced at the haustorial stage (Praz et al., 2018), suggesting that temporal expression patterns of the target gene could explain the limitation of loss of virulence to this developmental stage. However, we observed the same limited impact on virulence to the haustorial stage when we used transient HIGS to target *β2-tub*. Thus, demonstrating that this stage of development is highly relevant to control the pathogen via HIGS, independently of temporal target expression patterns. Similarly, it was speculated previously that stable HIGS lines could cause complete loss of virulence, instead our results suggest that the effect of HIGS on fungal virulence is quantitative by nature.

The primary target of this study *SvrPm3^*a*1/f1^* has first been characterized as a suppressor of the cell death triggered by the *Pm3* wheat resistance gene alleles (Bourras et al., 2015, 2019). By interfering with the recognition of cognate *AvrPm3* genes, *SvrPm3^*a*1/f1^* reduces the resistance spectrum of *Pm3* alleles. McNally et al. (2018) showed that the suppressor variant of *SvrPm3^*a*1/f1^* is present in wheat powdery mildew populations globally. We therefore conclude that combining HIGS of *SvrPm3^*a*1/f1^* with *Pm3* resistance alleles has the potential to increase the resistance spectrum of such wheat lines.

To our knowledge, this is the first comprehensive study of ck-RNAi in *Blumeria* based on stable transgenic HIGS lines. We conclude that ck-RNAi has great potential to complement current pest control strategies by e.g. broadening the resistance spectra of host resistance genes. We suggest that using ck-RNAi to tap naturally occurring RNA exchanges can provide new routes for crop improvement through genetic engineering as well as classical breeding.

## Materials and Methods

### Infection Tests

The United Kingdom *B.g. tritici* isolate Bgt_JIW2 (Wicker et al., 2013) and the Swiss *B.g. hordei* isolate K1 were maintained as described by Parlange et al. (2011) and Jordan et al. (2011), respectively. For high-density infection tests, inoculum for a 12 cm square plate was derived from three heavily infected 3 cm wheat leaves 10 dpi and applied using an infection tower of 25 cm height. Infected wheat leaves were scored macroscopically at 10 dpi. For low-density infection tests, inoculum was harvested at eight dpi from two mildly infected 2 cm wheat leaf segments, when the first mildew colonies start to sporulate, then dusted on leaf material in an infection tower. Infected wheat leaves were scanned six dpi using an HD-Scanner and “leaf area” and area covered by powdery mildew were estimated using Fiji^1^ based on color thresholding. The “infected leaf area” was calculated for each leaf by normalizing the infected area to the “leaf area”. The “relative infected leaf area” was further calculated by normalizing the “infected leaf area” to the mean infected leaf area of the non-transformed BW control from the same infection plate. For microscopy, high-density infection was used for two dpi samples and low-density infection for four and six dpi samples. Leave samples were destained (in 8% lactic acid, 16% glycerol, and 66% ethanol) and fungal structures were stained using coomassie blue. Aniline blue was used for papilla staining of a subset of two dpi samples, but since there were no observed effects on papilla formation it was omitted for the remaining samples. For each leaf, a minimum of 50 powdery mildew-wheat interactions, corresponding to an appressorium attacking an A- or B-type epidermal cell (Rubiales and Carver, 2000), were assessed for haustorium formation and shape, hyphae formation and number, and conidiophore formation and number.

### Construct Design and Cloning

The si-Fi software was used to select the donor sequences for the HIGS constructs (Lück et al., 2019). The candidate sequences were analyzed for putative off-targets in the respective donor genomes, *B.g. tritici* and *B.g. hordei*, and in the respective host genomes, wheat and barley (Mascher et al., 2017; Frantzeskakis et al., 2018; International Wheat Genome Sequencing Consortium, 2018; Müller et al., 2019). Additionally, CDS files of manually annotated *SvrPm3^*a*1/f1^* family members were used for target prediction in the isolate Bgt_JIW2. The *SvrPm3^*a*1/f1^*-RNAi sequence was amplified from genomic DNA of the Swiss *B.g. tritici* isolate Bgt_96224 (Stirnweis et al., 2014) using the primers BgtSvr-RNAi-F/R (Supplementary Table 2), cloned in reverse into the pIPKTA38 entry vector according to Douchkov et al. (2005) and transferred via Gateway cloning to the pIPKb007-RNAi vector (Himmelbach et al., 2007). The *β2-tub*-RNAi sequence was cloned into the pIPKTA30-RNAi vector from genomic DNA of isolate K1 using the primers Bgtβ2tub-RNAi-F/R (Supplementary Table 2).

### Transient HIGS

Transient HIGS was performed as described by Nowara et al. (2010). Here, the barley (*Hordeum vulgare*) cultivar “Golden Promise” and the wheat (*Triticum aestivum*) cultivar “Chancellor” were used. Three days after bombardment, leaves were infected with the barley powdery mildew isolate K1 and the wheat powdery mildew isolate Bgt_JIW2, respectively. The “haustorium index” represents the ratio of appressorium-forming spores that established a haustorium. Additionally, the presence of secondary hyphae was recorded. Results are derived from three independent experiments.

### Generation and Selection of Transgenic Events

Wheat transformation and transformant selection is described in Brunner et al. (2011). The *ubi:SvrPm3^*a*1/f1^*-RNAi transgene was excised from the pIPKb007 plasmid using *SfiI*. *SvrPm3^*a*1/f1^*-RNAi transgene presence was confirmed by PCR using the primers BgtSvr-RNAi-F/R (Supplementary Table 2). The selected T0 seedlings were transferred to the greenhouse and allowed to self. To select transgenic events for further analysis presence of both repeat sequences, copy number and transgene expression was assessed. Genomic DNA extraction is described in Brunner et al. (2011). Antisense repeat-specific primers pIPKb007-4/BgtSvr-RNAi-F and the sense repeat-specific primers pIPKb007-5/BgtSvr-RNAi-R were used to confirm presence of both repeats by PCR (Supplementary Table 2). Copy number was assessed by southern blotting 20μg *HindIII*-digested genomic DNA as described in Graner et al. (1990). For the probe, the repeat sequence was amplified using the BgtSvr-RNAi-F/R primers. Transgene expression was assessed by RT-PCR on cDNA. RNA was isolated as described in Brunner et al. (2011) with the alteration that leaves were collected in tubes and ground using glass beads. cDNA was synthesized from 500 ng RNA using the iScript^TM^ Advanced cDNA Synthesis Kit. The qRT-BgtBcg1F primer from Bourras et al. (2015) and BgtSvr-RNAi-R were used for amplification.

### RT-qPCR Assays

Biological replicates consist of first leaves of three 10-days-old seedlings which were pooled, immediately frozen in liquid nitrogen and stored at −80°C. RNA was extracted as described above with the alteration that infected samples were ground using metal beads. For target gene expression analysis, cDNA was synthesized as described above. Primers were designed to be specific to the fungal *SvrPm3^*a*1/f1^*, *Bgt_Bcg-6*, and *Bgt_Bcg-7* mRNAs (Supplementary Table 2). They were checked for specificity on plasmid DNA containing the transgene or either fungal target gene and on cDNA from infected non-transgenic and non-infected transgenic leaf material. Amplicons were verified by Sanger sequencing. *B.g. tritici gapdh* was used to normalize expression (Bourras et al., 2015). To further increase specificity, primer annealing was performed at 63°C and cDNA was quantified at 72°C. For consistency, these settings were used in all RT-qPCR analysis performed in this study. For transgene expression analysis, dsRNA and ssRNA were quantified according to Wang et al. (2018) with the following modifications: (1) RNA digested with *RNase If* was purified using the Promega SV Total RNA Isolation System, following the instructions in Promega Notes No. 86 2004 17, but using EconoSpin Micro Volume DNA/RNA Spin Columns. And (2) iScript^TM^ Advanced cDNA Synthesis Kit was used after hexamer incubation. qRT-BgtBcg1F/R primers from Bourras et al. (2015) were used to quantify transgene-derived transcripts. The wheat reference genes *Ta2291*, *Ta.6863*, and *Ta.25640* were used to normalize transgene expression (Supplementary Table 2; Giménez et al., 2011; Hurni et al., 2013). Results represent 3–4 biological replicates. The Kapa Sybr Fast qPCR Kit and the CFX384 Real-Time PCR Detection System were used.

### Semi-Field and Greenhouse Experiments

For the semi-field experiment we used the convertible glasshouse previously described in Romeis et al. (2007) and Boni et al. (2017) with a convertible roof programed to close in case of wind, rain, or cold. In this experimental setup the transgenic plants are retained from spreading to the environment in compliance with Swiss federal regulation. We planted a randomized setup of eight irrigated plots per event and wildtype BW, containing 10 plants each. Pre-germinated seeds were sown on March 29, 2018 into the 0.055 m^2^ central cylinders. More BW was sown as buffering plants around the central cylinders to reduce border effects. The soil was fertilized with 50 kg/ha P_2_O_5_, 85 kg/ha K_2_O, 40 kg/ha N, 11.7 kg/ha S, and 8.3 kg/ha Mg before sowing. At the start of stem elongation, test plants were reduced to 10 plants per cylinder and fertilized with 40 kg/ha N. The last fertilization with 40 kg/ha N was carried out before booting. Insecticides were applied as needed; weeding was done by hand. *B.g. tritici* infection was provided by natural inoculum. For AUDPC calculations, *B.g. tritici* disease was scored twice a week from beginning of May to end of June as described in Brunner et al. (2011). Representative leaves of adult plants were sampled per plot and infected leaf area was quantified as described above. Agronomical data was recorded per plot. 10 plants of each transgenic event and BW were also grown under greenhouse conditions. Data was recorded per plant. Plants were hand-harvested and threshed.

### Statistical Analyses

The statistical “exact and approximate test for proportions” in R was used to compare the *β2-tub-*RNAi construct results to the empty vector control. RT-qPCR experiment results were assessed for normal distribution and homogenous variance using the Shapiro–Wilk test and the Levene test, respectively. Based on this, one-way ANOVA or Welch ANOVA followed by the Tukey multiple pairwise comparison or the Kruskal–Wallis rank sum test followed by the Dunn’s *post hoc* test was used to test for statistical significance. Phenotyping results were assessed for normal distribution and homogenous variance using the Shapiro–Wilk test and the F-test, respectively. Based on this, the Wilcoxon rank sum, Welch *t*, or *t*-test was used to compare independent transgenic events to BW. All analyses were carried out using R software^2^.

## Data Availability Statement

All datasets generated for this study are included in the article/Supplementary Material.

## Author Contributions

LS, BK, and SB wrote the manuscript. LS, BK, SB, FP, and MM designed the experiments. LS, FP, GB, and GH performed the lab experiments. LS, EJ, JS, and RS performed the field experiments. LS, FP, TW, and SB performed the bioinformatics analyses. LS and AW performed the statistical analyses.

## Conflict of Interest

The authors declare that the research was conducted in the absence of any commercial or financial relationships that could be construed as a potential conflict of interest.

## Footnotes

1 https://imagej.net/Fiji

2 https://www.r-project.org/
