# Supplementary material for: Cross-Kingdom RNAi of Pathogen Effectors Leads to Quantitative Adult Plant Resistance in Wheat
Source: Front Plant Sci. 2020 Mar 10;11:253. doi: 10.3389/fpls.2020.00253 (PMC7076181; doi:10.3389/fpls.2020.00253)
Supplement: Supplementary file 1 [file Presentation_1.pdf]

Supplementary Material

1 Supplementary Figures and Tables

1.1 Supplementary Figures

>SvrPm3$^{a1/f1}$-RNAi
>SvrPm3$^{a1/f1}$
>Bgt_Bcg6
>Bgt_Bcg7

```
-100                                                                                   -11
|                                                                                        |
-----------------------------------CTCTCTGCAGCCCTGTTGATCCAGCCCGTTACTTCCCGAGCCAATCGACACAGAC
TCTTCATCTTCCCATCCACGTCCACTCGACACACTCTCTCTGCAGCCCTGTTGATCCAGCCCGTTACTTCCCGAGCCAATCGACACAGAC
TCTTCATCTTCCCATCCACGTCCACTCGACACACTCTCTCTGCAGCCCTGTTGATCCAGCCCGTTACTTCCCGAGCCAATCGACACAGAC
TCTTCATCTTCCCATCCACGCCCACTTGACACACTCTCTCTGCAGCCCTGTTGATCCAGCCTGTTACTTCCCGGGCCAATCGACACAGGC
...................................**************************.***********.**************.*
-10                                                                                     80
|                                                                                        |
TCGCTGCAAAATGAAGATTTTCTCCATCGCTGCCGTGGCGCTCCTTGTCGACATTTCAGTCTCATTTGACTTGATTGATGATGATGATAC
TCGCTGCAAAATGAAGATTTTCTCCATCGCTGCCGTGGCGCTCCTTGTCGACATTTCAGTCTCATTTGACTTGATTGATGATGATGATAC
TCGCTGCAAAATGAAGATTTTCTCCATCGCTGCCGTGGCGCTCCTTGTCGACATTTCAGTCTCATTTCACTTGATTGATGATGATGAAAC
TCGCAGCAAAATGATGTTTTTCTCCATCGCTGCCGTGGCGCTCCTTCTGGATATTTCAGTCTCATTTGAATTGATTGATGATGATAATGC
****.*********.*.*****************************.*.**.***************.*.***************.*..*
81                                                                                     170
|                                                                                        |
CTTCAGAAATAAACCCAAAATTCCTATTCTTAATAGGAAATTTGGGATGAAGTGTCAAACAAATACAATTTACAATGCAGAGACCATCAT
CTTCAGAAATAAACCCAAAATTCCTATTCTTAATAGGAAATTTGGGATGAAGTGTCAAACAAATACAATTTACAATGCAGAGACCATCAT
CTTTGGATATTATCCAAAAATTCCTATTCTTAACTCGAAATTCGGGATGAAGTGTCAAACAAATAACTTATACAATGCAGAGACTATCAA
ATCTAGAAATAATCCCCAAATTCCTGTTCTTAACTCGAAATTCGGGATGAAGTGTCATACAAATAACTTATACAATGCAGAGACTATCAA
.*...**.**.*.**..********.*******...******.**************.*******...*.**************.****.
171                                                                                    260
|                                                                                        |
GCAGAATCTTGACAAGGTGTATTCAAATAAG---CTACTTGCTCATCTGGAGCAAGGCAGCTTTACCCGTTTTCCTGGAGTAGTTTTCTA
GCAGAATCTTGACAAGGTGTATTCAAATAAG---CTACTTGCTCGTCTGGAGCAAGGCAGCTTTACCCGTTTTCCTGGAGTAGTTTTCTA
ACAGAAAGTTGATGAGGCGCATAATAATGTCCGATCTATTACTCGACTGGAACAAGGCACCTTTACCCGTTTTCCTGGAGTAGTTCTCTA
ACAGAATGTTGATGAAGCGCATAATAATGTCCGATTTATTACTCAACTGGAACAGGGCAGCTTTACCCGTTTTCCTGGAGTAGTTCTCTA
.*****..****..*.*.*.**...***..........**.***..*****.**.****.*************************.****
261                                                                                    350
|                                                                                        |
CCATTACTTCATAGGACAAAGTAACATTTCAGAACAAG----------------------------------------------------
CCATTACTTCATAGGACAAAGTAACATTTCAGAACAAGGTCATAATTACGCATCTGTGTCCTCATCATTTGCGGCCCACTAAATATTTTG
CAGTTACTTTATAGGACAAACTTACGATCTAGAACAAGGTCATAATTACATATCTTTGTCATCATCATTCGTGGCCCGCTAAATTTTTTG
CAGTTACTTCATAGGAAAAAGTTACGTTGCAGAACAAGGTCATAATTACACATCTGTCTCTTCATCATTCGCGGCCCGCTAAACT-TTTG
*..******.******.***.*.**..*..********....................................................
351                                                                                    440
|                                                                                        |
----GTTATTACCCCTCCAATTATATCATTGTCGACTCCCGCGGTGTGTTTGTGGCTGGGATGACATCAATCCCAGGCCTTTACCCAACG
CTAGGTTATTACCTCTCCAATTATATCATTGTCGACTCCCGCGGTGTGTTTGTGGCTGGGATGACATCAATCCCAGGCCTTTACCCAACG
CTAGATTATCACCCCTCCAATTATATCCTTATCGACTCCACCGGTCATTTGTGGCCGGGATGAAATCATGCGAAGCCAATGACCAAGTG
CTAGTTTATTACGCCTCCAATTACATCATTATCGACTCCTCCGGTGTGTTTGTGGCCGGGATGATATCAATCCCAGACAATGACAGAACG
.....****.**..*********.***.**.*********..***....********.*******.****..*..**.*..*.**..*..*
441                                                                                    530
|                                                                                        |
GCTTACAAAGTATGTAAGTTTACGGAGGAGACTGAGTTCGCCACTTACAGACGA------------------------------------
GCTTACAAAGTATGTAAGTTTACGGAGGAGACTGAGTTCGCCACTTACAGACGGCAATGACAAATCTATGTATCTAGCAATCAATCTTAT
CCCTACAGAGTATGTATGTTTACGGGGGGACTGATTACACCCGTTACGGATAATATTGATAACTCTATACATCTGGTAATCAATCTTAT
GCTTACAAAGTATGTAAGTTTACGGAGGGAACTGAATACGCCACTTATAGACAACTATGACAAATCTACACATTTGGCAATCAACCTTAT
.*.****.********.********.**..*****.*.*.**..***..**......................................
531                                                           594
|                                                              |
----------------------------------------------------------------
AATGGGTCTGGACATGAACAAACTGGCCCTCAACTACCTGTACGATCTCATATAGACCATTCAT
AATGGGTCTGGATATGAACAAACTGGCCCTCAACTACCTGTATGATCTCGTATAGACCATTCAT
AATGGGTCTGGGCTTGAACAAACTGGCCCTCAACTACCTGTATGATCTCATATAGACCGTTCAT
................................................................
```

Supplementary Figure 1. Alignment of the *SvrPm3*$^{a1/f1}$-RNAi repeat sequence with the *SvrPm3*$^{a1/f1}$, *Bgt_Bcg-6* and *Bgt_Bcg-7* genomic sequences of isolate Bgt_JIW2. Exons are highlighted in orange.

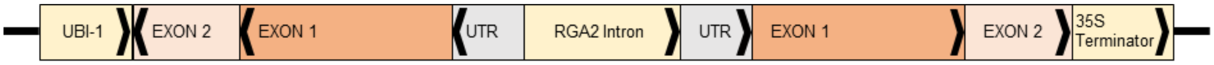

Supplementary Figure 2. The *SvrPm3*$^{a1/f1}$-RNAi transgene for stable wheat transformation consists of an inverted repeat of the *SvrPm3*$^{a1/f1}$-RNAi sequence. A *RGA2* wheat intron separates the two repeats to form the hairpin structure. Transgene expression is controlled by the maize ubiquitin promoter and the CaMV 35S terminator.

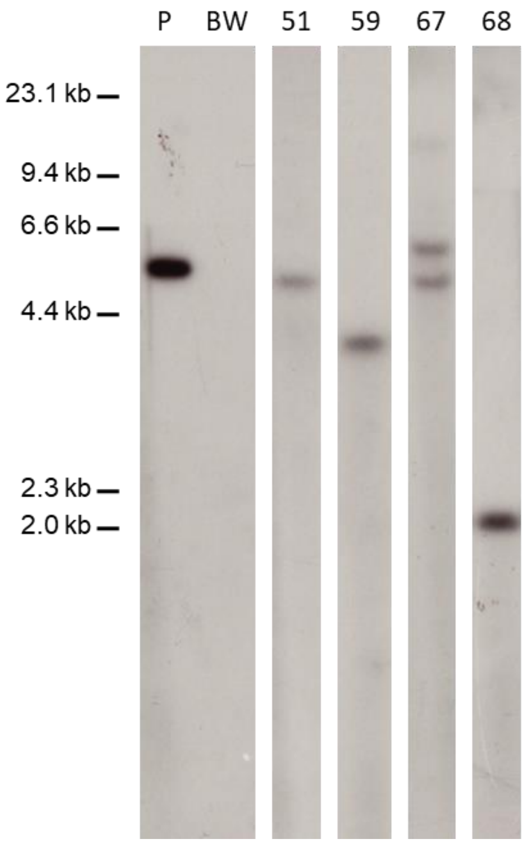

Supplementary Figure 3. Southern blot of *SvrPm3*$^{a1/f1}$-RNAi wheat lines. Genomic DNA was digested using *HindIII*, which cuts the transgene once upstream of the first repeat sequence. The ^{32}P-labeled probe binds to the *SvrPm3*$^{a1/f1}$ repeat sequence. Plasmid DNA served as a positive control (P), Bobwhite as a negative control (BW).

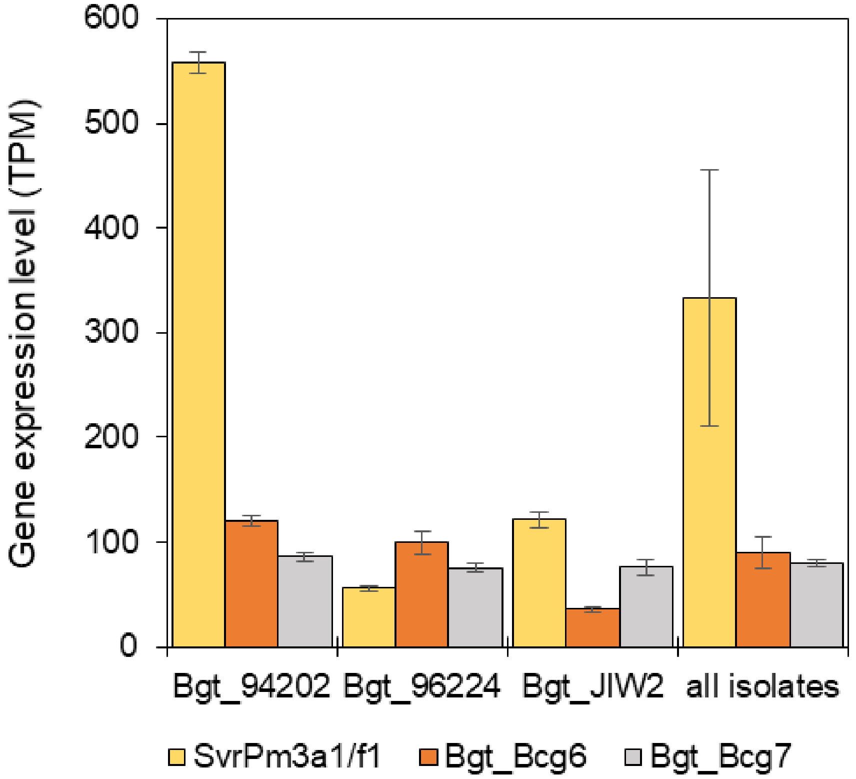

Supplementary Figure 4. Target gene expression in the three *B.g. tritici* reference isolates at two days post infection. RNAseq data has previously been published by Praz et al. (2018).

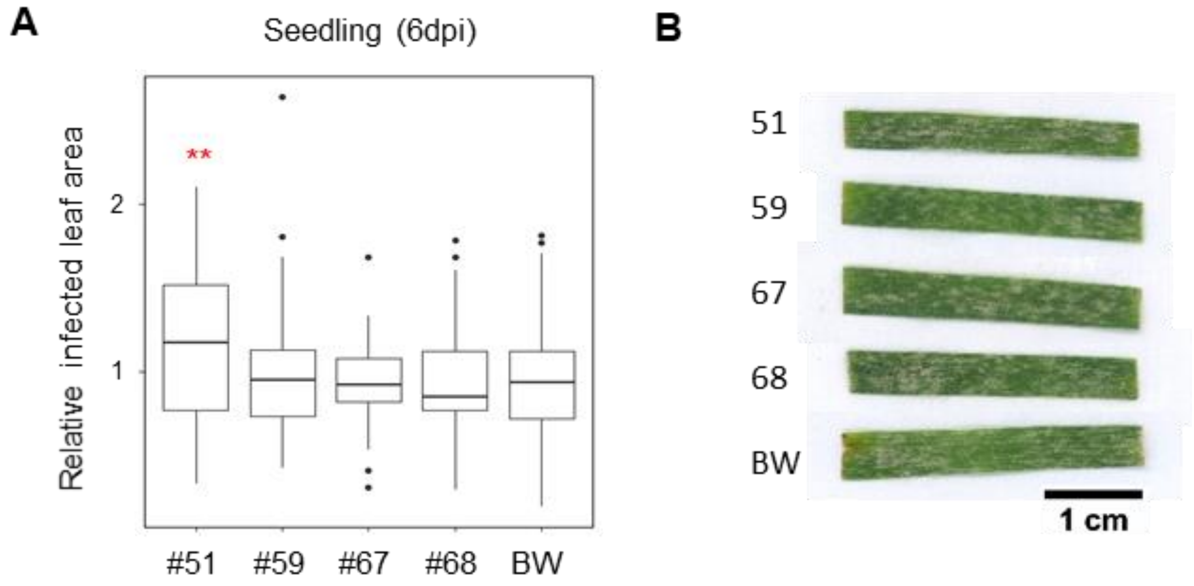

Supplementary Figure 5. *SvrPm3*$^{a1/f1}$-RNAi transgene does not reduce *B.g. tritici* disease symptoms on seedlings. Transgenic and wildtype (BW) wheat seedlings were infected with *B.g. tritici* isolate Bgt_JIW2 and infected leaf area was quantified six days post infection (dpi) using an HD scanner and Fiji imaging software. **(A)** Infected leaf area relative to the wildtype (BW). Data is derived from at least three independent experiments. **(B)** Leaf segments were selected to represent the median relative infected leaf area presented in (A). Pairwise comparison of transgenic events to wildtype BW was carried out using the two-sided Wilcoxon rank sum test. $^{**}p<0.01$

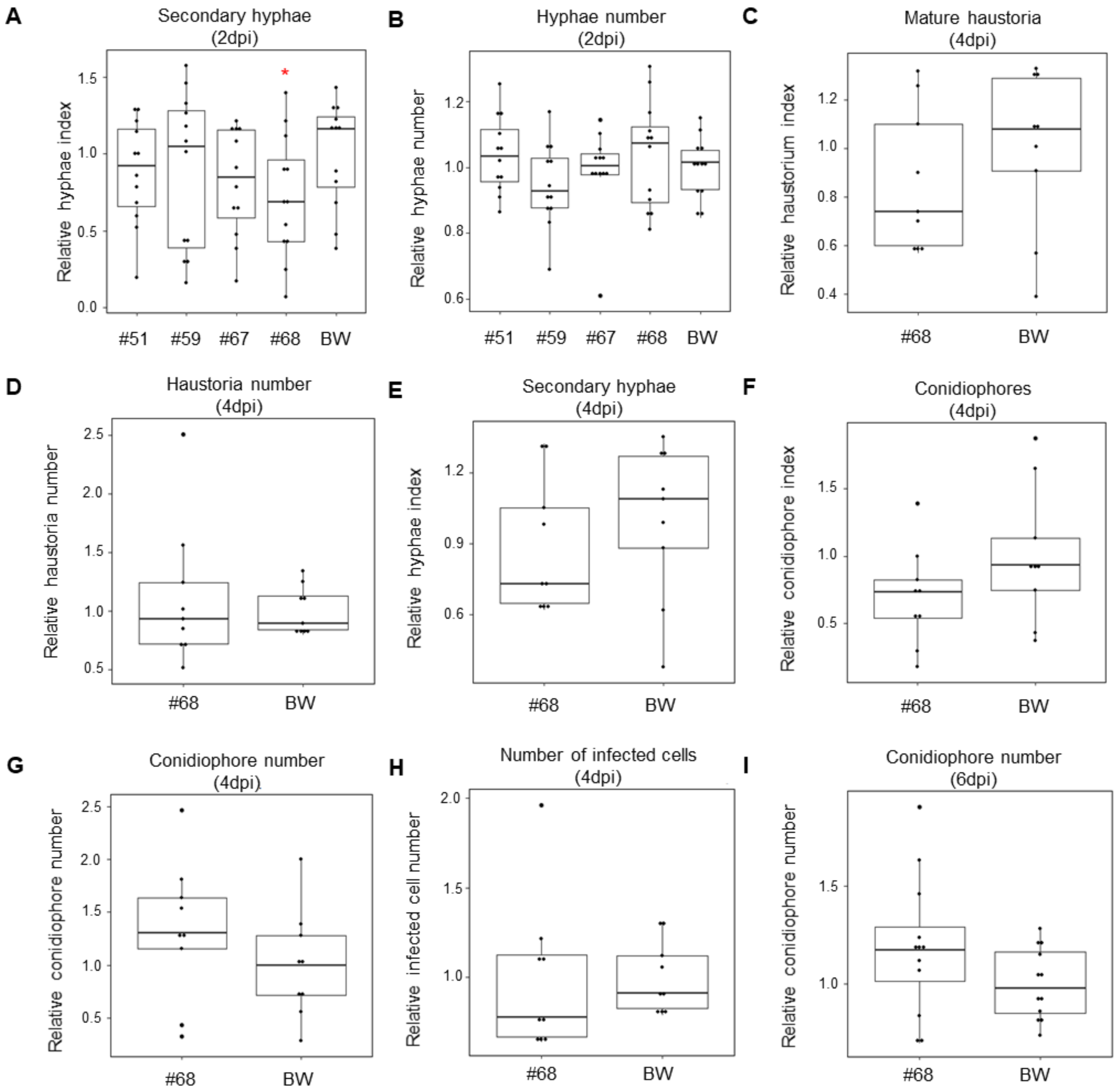

Supplementary Figure 6. Target effector silencing affects haustorium formation but no other stages of *B.g. tritici* development. *SvrPm3*$^{a1/f1}$-RNAi wheat seedlings were infected with *B.g. tritici* isolate Bgt_JIW2, and the transgene effect on virulence was scored at two-, four- and six-days post infection (dpi). **(A)** The effect on haustorium establishment at two dpi (Figure 2A) is accompanied by an equal effect on hyphae formation that results from the failure of initial infection. **(B)** No effect was observed on the number of hyphae per colony. **(C-H)** No effect on *B.g. tritici* virulence was observed at four dpi. **(G,I)** No effect on the number of conidiophores per colony was observed. Pairwise comparison of transgenic events to the wildtype BW was carried out using a one-sided t-test (A,C,E-G), Welch t-test (I) or a Wilcoxon rank sum test (B,D,H), according to the data distribution and variance. *$p<0.05$.

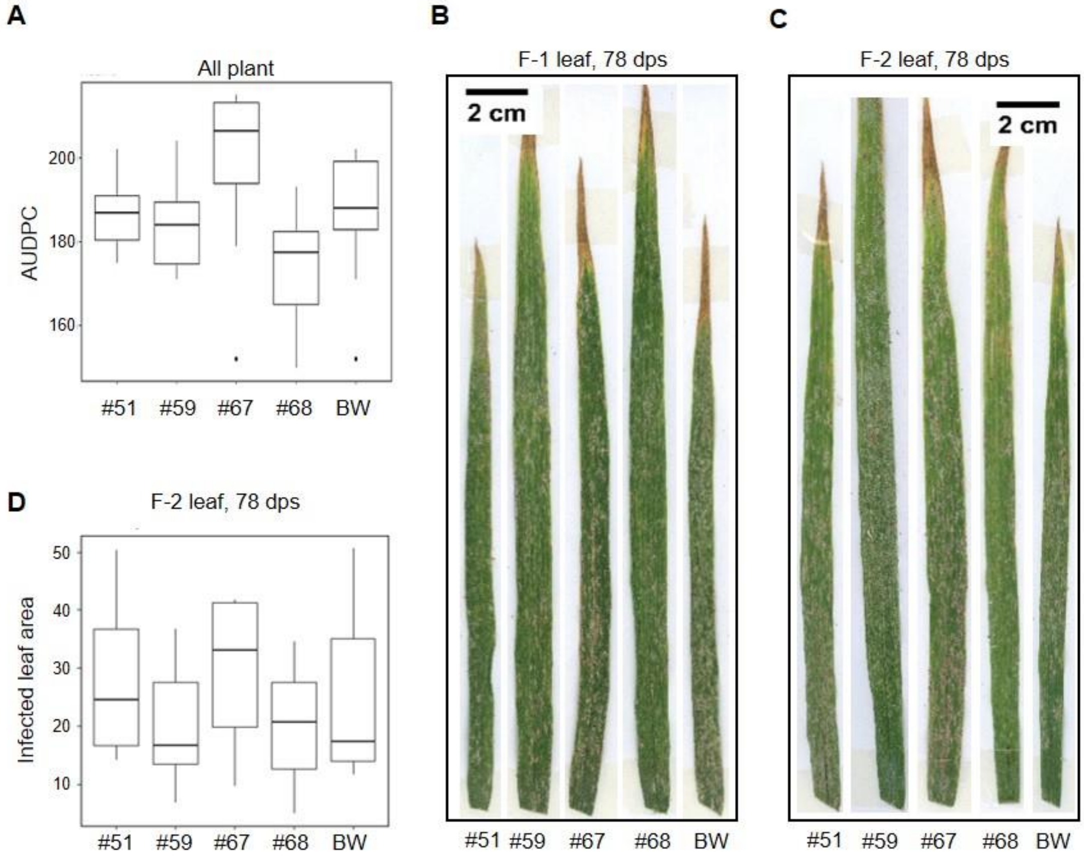

Supplementary Figure 7. *SvrPm3*$^{a1/f1}$-RNAi wheat lines show increase in resistance but only in the F-0 and F-1 leaves. Transgenic wheat lines and the wild-type control Bobwhite (BW) were exposed to environmental *B.g. tritici* infection in a semi-field trial. **(A)** *B.g. tritici* disease severity was monitored over time on the whole plant. The area under the disease progression curve (AUDPC) was calculated, revealing no significant effect when taking into account the whole plant and disease season. **(B-D)** Leaf coverage was measured on F-1 and F-2 leaves. **(B)** At 78 days post sowing (dps), the F-1 leaves show reduced disease symptoms on the events #59 and #68. **(C-D)** At 78 dps, the F-2 leaves show no significant reduction in disease symptoms. Pairwise comparison of transgenic events with wildtype BW were carried out using one-sided t-test (D) or Wilcoxon rank sum test (A) depending on the distribution of the data.

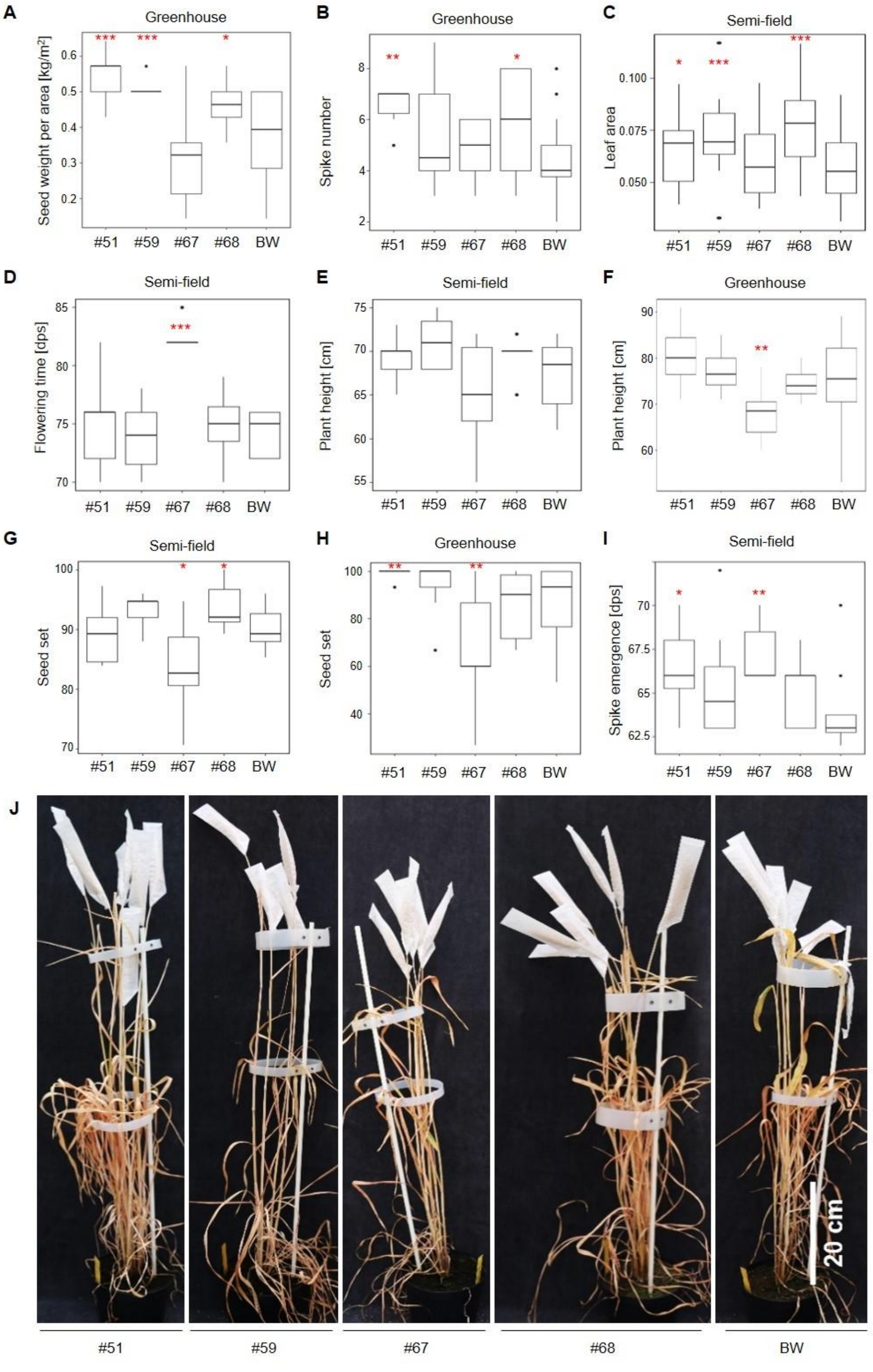
A
Greenhouse
Seed weight per area [kg/m²]
B
Greenhouse
Spike number
C
Semi-field
Leaf area
D
Semi-field
Flowering time [dps]
E
Semi-field
Plant height [cm]
F
Greenhouse
Plant height [cm]
G
Semi-field
Seed set
H
Greenhouse
Seed set
I
Semi-field
Spike emergence [dps]
#51
#59
#67
#68
BW
J
20 cm
#51
#59
#67
#68
BW

Supplementary Figure 8. *SvrPm3*$^{a1/f1}$-RNAi wheat lines display no negative effects on plant performance. Transgenic wheat lines and the wildtype control Bobwhite (BW) were exposed to environmental *B.g. tritici* infection in a semi-field trial. Transgenics and BW were also grown in the greenhouse where they were routinely treated with fungicides against *B.g. tritici* infection. **(A)** Under greenhouse conditions the transgenic lines #51, #59 and #68 showed an increased seed weight per area. **(B)** Events #51 and #68 had more spikes under greenhouse conditions. **(C)** Leaf area of the pooled representative F0, F-1 and F-2 leaf samples from the semi-field is significantly greater in the transgenic events #51, #59 and #68. **(D)** Event #67 flowered significantly later than other events and the BW control under semi-field conditions. **(E)** There was no significant effect on plant height under semi-field conditions. **(F)** Event #67 was growing shorter under greenhouse conditions. **(G)** The seed set of event #68 was fuller under semi-field conditions, while the seed set of event #67 was less full than BW. **(H)** The seed set of event #51 was fuller under greenhouse conditions, while the seed set of event #67 was less full than BW. **(I)** Spikes of events #51 and #67 emerged later under semi-field conditions. **(J)** Representative pictures of transgenic events and BW grown under greenhouse conditions. Pairwise comparison of transgenic events with wildtype BW were carried out using one-sided t-test (C,G), Welch t-test (F) or Wilcoxon rank sum test (A-B,D-E,H-I) depending on the distribution of the data. $^{*}p<0.05$; $^{**}p<0.01$; $^{***}p<0.001$.

Supplementary Table 1. Number of putative siRNAs generated by the *SvrPm3*$^{a1/f1}$-RNAi transgene that match the three target effectors in isolate Bgt_JIW2.

Target gene	All siRNAs	Efficient siRNAs
SvrPm3$^{a1/f1}$	430	105
Bgt_Bcg6	109	32
Bgt_Bcg7	37	10

Supplementary Table 2. List of PCR primer sequences

Primer ID	5’ to 3’ sequence
Bgtβ2tub-RNAi-F	CACCGCGAAGAGAAGCAGAGGGAT
Bgtβ2tub-RNAi-R	TGCCATCATTCGATCAGGAA
BgtSvr-RNAi-F	CTCTCTGCAGCCCTGTTGAT
BgtSvr-RNAi-R	TCGTCTGTAAGTGGCGAACTC
pIPKb007-5	TCCAGCTTCCGAGTCCTGAAAAA
pIPKb007-4	CTGCAGGAATTCAAGCTTACG
qRT-BgtSvr-F	ATCCCAGGCCTTTACCCAAC
qRT-BgtSvr-R	AGGGCCAGTTTGTTCATGTC
qRT-BgtBcg6-F	TGACCAAGTGCCCTACAGAG
qRT-BgtBcg6-R	CAGGTAGTTGAGGGCCAGTT
qRT-BgtBcg7-F	TTATCGACTCCTCCGGTGTG
qRT-BgtBcg7-R	AGTGGCGTATTCAGTTCCCT
qRT-Ta.25640-F	CAGAGGAGAGATCACCGGAT
qRT-Ta.25640-R	GTCGAGCTTGAGGGTTACAG

2 Supplementary Notes

Supplementary Note 1. *SvrPm3$^{a1/f1}$*-RNAi putative off-target on wheat chromosome 6A

si-Fi predicted four *SvrPm3$^{a1/f1}$*-RNAi construct derived siRNAs that could potentially target wheat chromosome 6A. Though, all four are predicted by si-Fi to be inefficient in directing gene silencing. The predicted 30-nt off-target locus was not annotated previously and lies inside a putative pseudogene (Supplementary File 2). The sequence surrounding the off-target shows homology at the protein level to hypothetical proteins from *Triticeae*, rice and Brachypodium. Protein structure prediction identified homology to the RRM domain of the human RNA binding (adp-2 ribose) polymerase family (member 10) (Supplementary File 2). However, due to the presence of multiple frameshifts in the predicted CDS, this region is unlikely to contain a functional gene.

3 Supplementary Files

Supplementary File 1. Sequence of the *β2-tub*-RNAi repeat used in transient HIGS to silence *β2-tubulin* in *B.g. hordei* and *B.g. tritici*

Supplementary File 2. *SvrPm3$^{a1/f1}$*-RNAi putative off-target on wheat chromosome 6A sequences
