# Supplementary material for: Cross-Kingdom RNAi of Pathogen Effectors Leads to Quantitative Adult Plant Resistance in Wheat
Source: Front Plant Sci. 2020 Mar 10;11:253. doi: 10.3389/fpls.2020.00253 (PMC7076181; doi:10.3389/fpls.2020.00253)
Supplement: Supplementary file 2 [file Data_Sheet_1.pdf]

Supplementary File 1

>Beta2-tub-RNAi repeat sequence
GCGAAGAGAAGCAGAGGGATGTGACTGTCTTCAGGGGTTTCAAATAA
CACATTCTCTTGGGGGTGGTACAGGTGCCGGTATGGGTACGTTATTAA
TTTCAAAAATCCGGGAAGAATTCCCTGATCGAATGATGGCA
